# Patterning the mechanical properties of hydrogen silsesquioxane films using electron beam irradiation for application in mechano cell guidance

**DOI:** 10.1016/j.tsf.2010.10.054

**Published:** 2011-01-03

**Authors:** Mathieu Lanniel, Bingrui Lu, Yifang Chen, Stephanie Allen, Lee Buttery, Phil Williams, Ejaz Huq, Morgan Alexander

**Affiliations:** a Laboratory of Biophysics and Surface Analysis, School of Pharmacy, University of Nottingham, University Park, Nottingham NG7 2RD, UK; b Rutherford Appleton Laboratory, Harwell Science and Innovation Campus, Didcot, OX11 0QX, UK

**Keywords:** Hydrogen silsesquioxane, Mesenchymal stem cells, Electron-beam curing, Atomic force microscopy, Plasma polymerization

## Abstract

Hydrogen silsesquioxane (HSQ) is a material with the potential for studying the effect of surface stiffness on stem cell differentiation. Here, the effects of electron beam dose on the topography and the mechanical properties of HSQ obtained with or without trimethylamine (TMA) development are characterised by atomic force microscopy imaging and indentation. A correlation between the surface stiffness (uniform across the sample) and electron beam exposure is observed. Surface roughness of HSQ samples developed in TMA decreases exponentially with increasing electron beam exposure. Surface coating with plasma polymerised allylamine (ppAAm) leads to an overall decrease in stiffness values. However, the increase in surface stiffness with increasing electron beam exposure is still evident. The ppAAm coating is shown to facilitate human mesenchymal stem cell adhesion.

## 1 Introduction

Understanding the effects of the cellular microenvironment in modulating stem cell morphology and lineage specification is a current area of intense research. Many studies have focussed on the effects of surface chemistry on stem cell differentiation, but recently surface stiffness has also been recognized as an important factor for directing stem cell fate. Studies on mesenchymal stem cells have revealed that these cells commit to different lineages according to the stiffness of the substrate used for cell culture [1,2]. This phenomenon is thought to be caused by a cellular mechano-transducer(s) that generates signals based on the force that the cell must generate to deform the substrate on which they are adhered [1]. Biomaterials with tunable elastic modulus have therefore potential application in controlling stem cell lineage. Cell attachment to materials has also been shown to depend on the surface chemistry and topography of the material [3–6]. It has been noted that amino groups are particularly favourable for the immobilisation of adhesive proteins, which promote the adhesion of certain cells [5,7]. This study explores the potential of hydrogen silsesquioxane (HSQ) coated with plasma polymerised allylamine (ppAAm) as a material for studying the effect of surface stiffness on stem cell differentiation.

HSQ is an inorganic polymer with the general formula (HSiO_3/2_)_2n_. In 1998, Namatsu et al. [8] discovered that HSQ could be used as a negative electron beam resist combining high resolution at a moderate sensitivity with minimum line edge roughness [9]. The chemical structure of HSQ before curing can be described as one of “caged oligomers”. During curing, the silicon hydrogen (Si–H) bonds (that are weaker than silicon oxygen (Si–O) bonds) are broken and converted to silanol groups (Si–OH) in the presence of absorbed moisture in the film. These silanol groups are unstable and condense to break the caged molecule and form a linear network. After electron beam exposure, HSQ has an amorphous structure similar to silica that is relatively insoluble in alkaline hydroxide developers. The HSQ material containing less than the critical ratio of network/cage structure is dissolved and removed during development [10].

Two essential parameters for the characterization of electron beam resists are contrast and sensitivity. The contrast is defined as the slope of the contrast curves obtained by plotting the normalized remaining resist thickness after development as a function of the logarithm of the electron dose. The sensitivity is defined, for a negative resist, as the dose at which all the exposed resist remains after development and for a positive resist, as the dose at which all the exposed resist is removed after development. Previous studies have shown that many parameters during the processing of HSQ (baking temperature, development concentration, development temperature and electron beam exposure) have a high influence on contrast and sensitivity. This affects the mechanical and topographical properties of the resist [9,11–14].

Georgiev et al. [9] found that HSQ samples baked at 220 °C before electron beam curing and development in tetramethylammonium hydroxide (TMAH) had much higher roughness values than samples prebaked at 90 °C. In comparison, the TMAH concentration had a very weak influence on the resulting HSQ roughness. For all the conditions, the roughness decreased with increasing electron beam exposure and levelled off at about 1 nm at the dose where the corresponding contrast curve saturates. The differences in roughness were not present on the HSQ samples before the development step. These observations were explained by the fact that thermal processing induces bond scission and recombination favouring the transition from the cage structure to a network structure [15]. The pre-bake step can therefore be considered as a pre-exposure before the electron beam curing although with a lower energy transfer. At 220 °C prebake, network clusters are formed, which are removed during the development step in the case of low electron beam exposure, leading to a higher roughness than on the corresponding samples baked at 90 °C. The study concluded that a combination of a strong developer with low baking temperature is favourable for achieving the highest possible resolution using HSQ as a negative tone electron beam resist. Another study by Chen et al. [14] found that increasing the temperature used for the development step allowed more efficient removal of unexposed HSQ, thereby increasing contrast. Other studies have focussed on the effect of HSQ curing on the resulting mechanical properties of the resist. Toivola et al. [12] studied infra-red spectra obtained on HSQ samples exposed to different temperatures for thermal curing. They showed a progressive decrease in the number of Si–H bond and a progressive increase in the number of Si–O bond, associated with the network structure, with increasing curing temperatures. These changes of cross linking ratios were also observed on the Young's modulus of the HSQ samples, with an increase from 6.4 GPa to 13.7 GPa measured by nanoindentation when the curing temperature was changed from 375 °C to 450 °C. For even higher temperatures, Siew et al. [16] found an excessive loss of hydrogen leading to a collapse of the porous network structure and to a denser HSQ structure. Liou et al. [17] found a linear relation between elastic modulus and curing temperature using a range from 350 °C to 800 °C, with modulus values comprised between 5 GPa and 80 GPa. They correlated this change in modulus with the Si–H/Si–O ratio. At small Si–H/Si–O ratios (i.e. at high crosslinking obtained with high curing temperatures), the elastic modulus increases much faster because densification of the network structure causes larger changes in the mechanical properties. In contrast to these detailed studies of the dependence of the mechanical properties of HSQ with curing temperature, we know of no studies investigating the effect of electron beam exposure on the elastic modulus.

The present study covers a range of electron beam exposures from 7 μC cm^−2^ to 5000 μC cm^−2^ for undeveloped HSQ samples and those developed in trimethylamine (TMA). The HSQ samples used consist of arrays of 100 μm × 100 μm “pads” exposed to increasing electron beam doses. In order to improve cell adhesion to the sample surface while achieving uniform chemistry across the samples, ppAAm coatings were deposited on the HSQ samples. Plasma polymers can be deposited as ‘conformal’ coatings onto any solid with only minimal change in topography, and they are useful to prepare a large variety of surface chemistries [18–20]. ppAAm coatings are known to contain nitrogen-containing groups in relatively high concentrations and have been shown to promote adhesion of certain cells [21–23]. Elastic modulus of the HSQ samples was characterized using atomic force microscopy (AFM) nanoindentation and torsional tapping mode imaging [24]. Torsional tapping mode is a recently developed AFM mode that provides nanoscale quantitative material property mapping of adhesion, stiffness, dissipation, peak force, and average force. We use this to reveal information on the distribution of the modulus values across the HSQ samples exposed to the two extreme electron beam doses. The effect of electron beam exposure on surface roughness was also studied with and without a development step. It was determined that mesenchymal stem cells adhered to HSQ coated with plasma polymerized allylamine. The ability to pattern the modulus of HSQ using electron beam exposure provides great opportunities for the control of stem cell differentiation on the length scales accessible to electron beam writers. This exciting finding has potential application in the emerging fields of regenerative medicine and stem cell therapy.

## 2 Experimental details

### 2.1 Materials preparation

The HSQ samples used in this study consisted of arrays of 100 μm × 100 μm “pads” exposed to increasing electron beam doses. To obtain these arrays, commercial hydrogen silsesquioxane solution (Fox-24, Dow Corning Corporation, Midland, MI) was spin-coated at 4000 rpm onto silicon wafers. The samples were then put in vacuum of 1 kPa for 5 min. Electron beam exposure of HSQ was carried out by a high resolution vector beam writer (VB6 HR) from Leica Microsystems at an acceleration voltage of 100 keV within a wide range of doses. For the developed HSQ array, the dose ranged from 6 μC cm^−2^ to 2000 μC cm^−2^ across 100 of the HSQ pads. However, after development, only 80 pads exposed to doses from 19.4 μC cm^−2^ to 2000 μC cm^−2^ remained. For the undeveloped sample, the electron beam exposures ranged from 7 μC cm^−2^ to 5000 μC/cm^2^ across 100 HSQ pads. The thickness of the HSQ was approximately 620 nm, as measured by ellipsometry. One sample was then developed for 2 min in the aqueous base developer TMA to remove unexposed HSQ material.

To allow cell attachment, the HSQ arrays were coated with ppAAm in a plasma deposition reactor. The arrays were etched with oxygen for 5 min at working pressure of 40 Pa and the allylamine monomer was then fed in the reactor chamber to a pressure of 40 Pa. The coating process was done using a glow discharge power of 20 W for 5 min. The thickness of the plasma coating was measured by ellipsometry and estimated at 30 nm using the Cauchy model.

The effect of plasma polymerised allylamine film thickness on the Young's modulus of silicon was investigated. After oxygen etching silicon wafers for 5 min at a working pressure of 40 Pa, a range of thickness of ppAAm was deposited at a power of 20 W and at a working pressure of 40 Pa for varying times. The thickness of the plasma coatings on the silicon wafers was measured by ellipsometry and estimated using the Cauchy model.

### 2.2 Materials characterization

The Young's modulus and the root-mean-square (RMS) roughness values were determined using a Veeco Dimension 3000 Atomic Force Microscope (Veeco, Sunnyvale, CA) operated with Tap300Al probes (Budget Sensors). The HSQ films were indented by less than 10% of their thickness to avoid the measurement being affected by the substrate. A constant load of 4 μN was applied for all force measurements. For the HSQ surface roughness measurements, squares of 5 μm × 5 μm have been scanned with a 512 by 512 grid and a frequency of 1 Hz per line.

Torsional tapping imaging is a recently developed AFM mode allowing obtaining measurement of physical properties of surfaces while imaging via tapping mode. For this, the force interaction between the tip and surface are reconstructed during tapping mode imaging by measuring the torsional amplitude of the AFM cantilever at higher harmonic frequencies of the tapping mode drive frequency. This mode requires the use of specially designed probes where the tip is offset to one side of the cantilever. The torsional tapping imaging was performed on uncured HSQ as well as on HSQ pads exposed to the extreme electron beam doses for the undeveloped array (7 μC cm^−2^, 2000 μC cm^−2^ and 5000 μC cm^−2^) and the developed array (19.4 μC cm^−2^ and 2000 μC cm^−2^) using HarmoniX probe silicon cantilevers (Veeco, Santa Barbara, CA) with a spring constant of 4 N m^− 1^, vertical resonance frequency of 55 kHz. The stiffness channel was calibrated using a reference surface of polystyrene and low density polyethylene thin film for imaging, knowing that the stiffness of the polystyrene sample is 1.6 GPa.

### 2.3 Cell culture

Human Mesenchymal Stem Cells (hMSC) derived from the bone marrow were cultured in normal growth media (TCS cellworks Ltd). All cells were used at passage number 5, were expanded on gelatine, were plated on the HSQ arrays at 1000 cells/cm^3^ and were cultured in low-glucose Dulbecco's modified Eagle's medium supplemented with 20% fetal bovine serum, 50 μg.mL^−1^ streptomycin, and 50 units mL^−1^ penicillin for 7 days. Medium was changed every 2 days.

## 3 Results

**Fig. 1:**
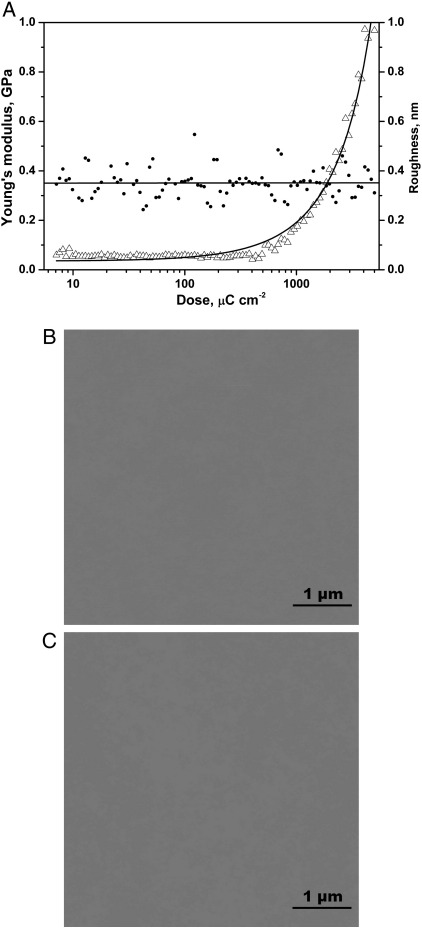
A. Young's modulus (Δ) and RMS roughness (∙) of the undeveloped HSQ array as a function of electron beam exposure. B–C. 25 μm^2^ AFM images (Z-scale: 1 μm) of undeveloped HSQ pads exposed to an electron beam dose of 7 μC cm^−2^ (B) or 5000 μC cm^−2^ (C).

**Fig. 2:**
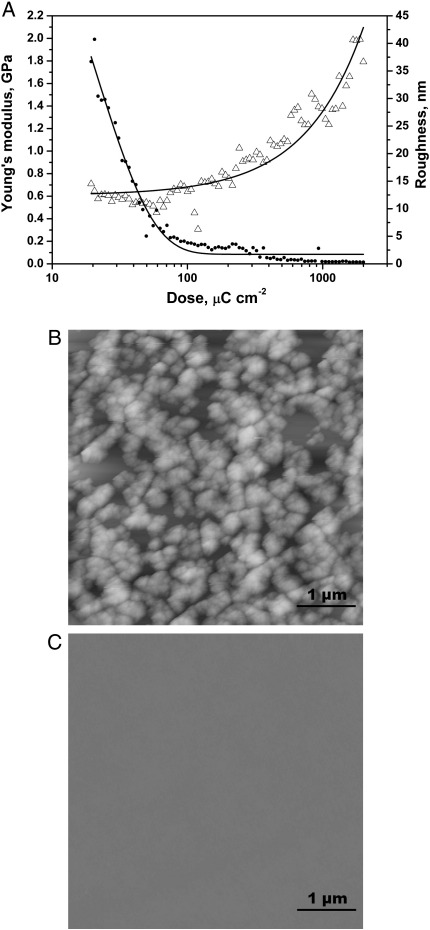
A. Young's modulus (Δ) and RMS roughness (∙) of the developed HSQ array as a function of electron beam exposure. B–C. 25 μm^2^ AFM images (Z-scale: 1 μm) of developed HSQ pads exposed to an electron beam dose of 19.4 μC cm^−2^ (B) or 2000 μC cm^−2^ (C).

In order to study the electron beam exposure dose dependence of the HSQ stiffness and roughness and the effects of development by TMA on these properties, two HSQ arrays, one developed in TMA and one undeveloped, were subjected to force measurements and the same area of each HSQ pad was imaged using tapping mode AFM. The results for the undeveloped HSQ array are summarized in Fig. 1, and the ones for the developed array are summarized in Fig. 2.

For both HSQ arrays, the Young's modulus values increased with the electron beam dose. For the undeveloped HSQ array, the modulus values increased from 0.06 GPa to 1 GPa for an increase in dose range from 7 μC cm^−2^ to 5000 μC cm^−2^. For the exposure range used for the developed HSQ array (19.4 μC cm^−2^ to 2000 μC cm^−2^), the modulus values increased from 0.5 GPa to 2 GPa. Comparing the modulus values at equal electron beam exposures for both samples it appears that developed HSQ is stiffer than the undeveloped HSQ. For an exposure of 20 μC cm^−2^, the developed sample has a modulus of 0.7 GPa while the undeveloped sample has a far lower modulus of 0.06 GPa. At 2000 μC cm^−2^, the modulus values for developed and undeveloped samples were 2 GPa and 0.4 GPa, respectively. The observation that this difference in stiffness is more pronounced at lower electron exposures, suggests that the presence of partially cured material in the undeveloped samples results in a lower stiffness. For both HSQ arrays, no effect is seen on the Young's modulus for slight changes in dose at low electron beam doses. However at doses higher than 330 μC cm^−2^, a far greater sensitivity of the Young's modulus to electron beam dose was observed.

The RMS roughness was calculated using tapping mode AFM images obtained on each pad for both the developed and undeveloped arrays. Although the RMS deviation of roughness does not provide information about the lateral dimensions of roughness but only about the vertical magnitude, it is the most widely used parameter in the field of lithography and can easily be deduced from AFM images. For the developed HSQ array, an exponential decrease of roughness is observed as a function of the logarithm of electron beam exposure. Roughness values range from 40 nm for the lowest exposure (19.4 μC cm^−2^) to 0.3 nm for the highest exposure (2000 μC cm^−2^). This variation of RMS roughness is not observed on the undeveloped sample where the roughness values are constant (between 0.2 nm and 0.6 nm) and correspond to the roughness values obtained for high electron beam exposures on the developed sample. This suggests that the dose dependent roughness observed on the developed sample is caused by removal of uncured HSQ material on the surface of the HSQ pads during the development step.

**Fig. 3:**
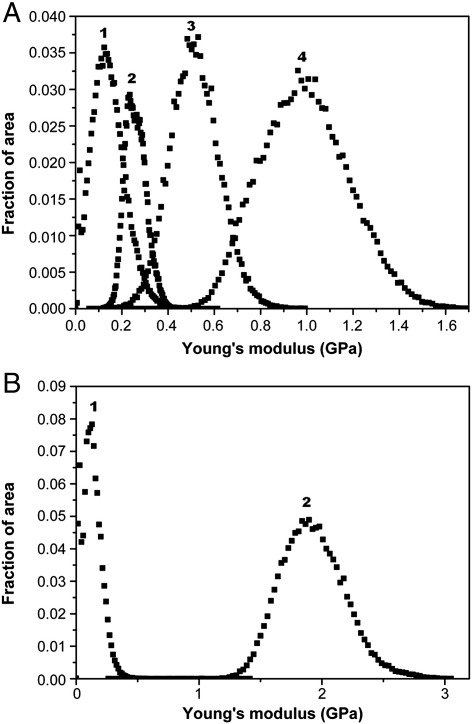
Histograms of Young's modulus values obtained by Harmonix imaging on a 25 μm^2^ area on developed and undeveloped HSQ. A. Young's modulus values obtained on the undeveloped HSQ array for (1) uncured HSQ and pads exposed to (2) 7.1 μC cm^−2^, (3) 2000 μC cm^−2^ and (4) 5000 μC cm^−2^ electron beam dose. B. Young's modulus values obtained on the developed HSQ array for pads exposed to (1) 20.4 μC cm^−2^ and (2) 2000 μC cm^−2^ electron beam dose.

Torsional tapping imaging was carried out on both HSQ arrays for the extreme electron beam exposures on 25 μm^2^ areas as well as on uncured HSQ. This imaging mode allows mechanical properties to be determined at each pixel of an image. The results for the Young's modulus imaging are shown in Fig. 3 in the form of a frequency versus modulus plot. The histogram for the uncured HSQ shows uniform stiffness values with a mean of 0.14 GPa. For the undeveloped HSQ array, the pad exposed to low doses of electron beam has a mean stiffness of 0.26 GPa and shows a uniform distribution across the area studied. These values are higher than the ones obtained using AFM nanoindentation where low electron beam exposure lead to Young's modulus values of about 0.06 GPa. For high electron beam exposures, the histogram of the Young's modulus values shows a higher variation in stiffness values than the peaks obtained at low exposures. For 5000 μC cm^−2^ electron beam exposure, the peak of the distribution is centered at 1 GPa, which is consistent with the previous force measurements obtained on this sample. For HSQ exposed to 2000 μC cm^−2^, the distribution of Young's modulus is centered on 0.53 GPa, which is close to the value of 0.4 GPa obtained by AFM nanoindentation. For the developed HSQ array, at the lowest electron beam exposure, the stiffness values are centered at 0.2 GPa, although a smaller peak is also observed at about 0.02 GPa. This might be caused by the high roughness observed on developed HSQ at low electron beam doses. For the highest electron beam exposure (2000 μC cm^−2^), the peak is centered at 1.89 GPa, close to the value of 2 GPa measured by AFM nanoindentation.

**Fig. 4:**
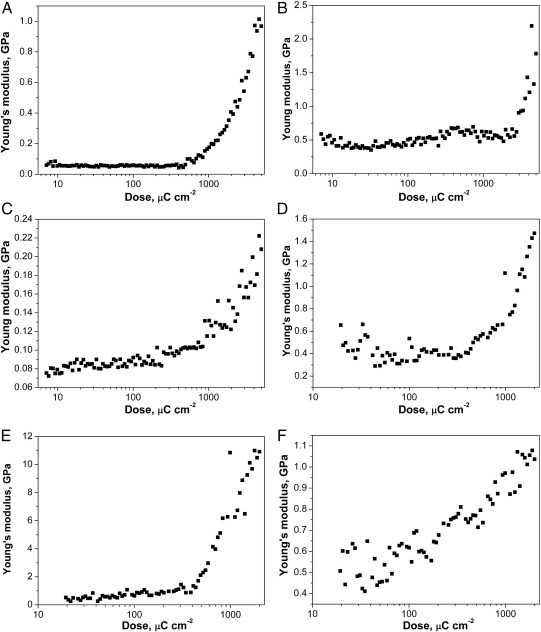
A, B, C. Young's modulus values as a function of electron beam exposure for the undeveloped HSQ array, (A) without treatment, (B) after oxygen plasma treatment and (C) after ppAAm deposition. D, E, F. Young's modulus values as a function of electron beam exposure for the developed HSQ array, (D) without treatment, (E) after oxygen plasma treatment and (F) after ppAAm deposition.

**Fig. 5:**
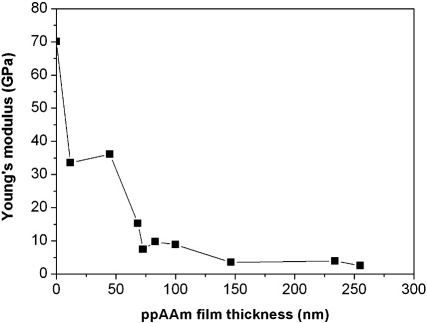
Young's modulus of ppAAm coated silicon versus ppAAm film thickness determined by AFM nanoindentation.

To improve cell attachment to HSQ, the arrays were exposed to oxygen plasma followed by deposition of ppAAm. The effect of these treatments on the Young's modulus of HSQ was studied using AFM nanoindentation. The results for the undeveloped and developed HSQ arrays are shown in Fig. 4. For both arrays, the oxygen plasma treatment leads to a general increase in Young's modulus values but the difference of stiffness between pads exposed to low or high electron beam doses is still visible. For the undeveloped array, the range of stiffness obtained increases from 0.06 GPa to 0.5 GPa for the lowest electron beam exposures and from 1 GPa to 2.3 GPa for the highest electron beam exposures. For the developed array, an increase in stiffness is also observed but the effect of oxygen treatment is more important for pads exposed to high electron beam doses. For the lowest electron beam exposures, the Young's modulus increases from 0.3 GPa to 0.5 GPa and for the highest electron beam exposures, the Young's modulus increases from 1.5 GPa to 11.5 GPa. The increase in stiffness observed is likely to be caused by additional cross linking of the HSQ structure caused by the oxygen plasma. Allylamine deposition after oxygen plasma treatment leads to a decrease in the measured Young's modulus values for both arrays. For the undeveloped HSQ array, the stiffness ranges from 0.13 GPa to 0.45 GPa for the pads exposed to the lowest and highest electron beam doses, respectively. For the developed HSQ array, the stiffness ranges from 0.4 GPa to 1.1 GPa for the pads exposed to the lowest and highest electron beam doses, respectively. The indentation depth of the AFM tip varies from 52 nm for the softest HSQ pads to 33 nm for the hardest one. As the ppAAm thickness is approximately 30 nm, the measured Young's modulus is obtained as a combination of the soft ppAAm film and the harder HSQ surface underneath. Since ppAAm is softer than HSQ, ppAAm coating leads to a decrease in the modulus measured by AFM. The analysis depth of the experiment has been characterised by examining a variety of coating thicknesses on silicon wafers and is presented in Fig. 5. The results of the AFM nanoindentation show that the measured Young's modulus values of coated silicon wafers progressively decrease with increasing ppAAm film thickness, from 70 GPa for uncoated silicon to about 1 GPa for 255 nm ppAAm film thickness. The measured Young's modulus values represent therefore a combination of the deposited ppAAm film and the underlying silicon substrate. The indentation depth is comprised between 2 nm for uncoated silicon to 68 nm for the silicon wafer coated with 255 nm thick ppAAm film. Examination of Fig. 5 indicates that the Young's modulus reaches a constant value of 2.5 GPa above a ppAAm thickness of 150 nm. This suggests that the analysis depth of AFM nanoindentation on the ppAAm coatings is between 75 and 150 nm.

**Fig. 6:**
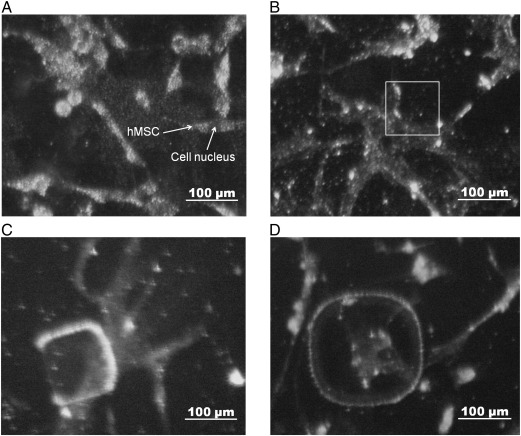
Human mesenchymal stem cells cultured on the HSQ array coated with plasma polymerized allylamine after 7 days of culture. A, B. hMSC cultured on undeveloped HSQ. (A) Uncured HSQ, (B) HSQ exposed to 5000 μC cm^−2^ electron beam dose. hMSC cultured on developed HSQ pads exposed to (C) 19.4 μC cm^−2^ electron beam dose and (D) to 1 973 μC cm^−2^ electron beam dose.

Human mesenchymal stem cells showed a very limited adhesion to uncoated HSQ. Plasma polymerised allylamine was used to amino functionalise the surface to improve adhesion. The cells were found to be able to attach to the ppAAm coated HSQ surfaces for a long culture time (7 days), (Fig. 6**)**. The cells were initially small and round shaped but developed increasingly branched and polygonal shapes, showing good adhesion to the surface. No significant variation of cell morphology was observed as a function of the stiffness variation in HSQ in these experiments.

## 4 Discussion

In the present study, it was observed that a range of Young's modulus values of HSQ could be obtained by varying electron beam exposure dose. The Young's modulus values ranged from 0.06 GPa to 1 GPa for an undeveloped HSQ array (dose range: 7–5000 μC cm^−2^) and from 0.7 GPa to 2 GPa for a developed array (dose range: 19.4–2000 μC cm^−2^). Previous studies determined Young's modulus values in the GPa range for HSQ: Liou et al. [17] used HSQ samples that were thermally cured using temperatures between 350 °C and 800 °C for 1 h. A linear correlation was found between the Young's modulus of HSQ and the curing temperature used, with Young's modulus values ranging from 5 to 85 GPa for the lowest and highest temperatures, respectively. Another study by Toivola et al. [12] using thermal curing for 1 h with temperatures ranging from 375 °C to 450 °C also observed an increase in Si–O/Si–H ratio and Young's modulus of HSQ with increasing curing temperature. Modulus values ranged from 6.4 GPa for 375 °C curing temperature to 13.7 GPa for 450 °C, as measured by nanoindentation. These results are in agreement with the previous study by Liou et al. [17] and in both studies, the Young's modulus values obtained were higher than the ones from the present study. This could be caused by the difference between the curing methods used (electron beam or thermal curing). This could also be caused by the method used for the determination of the Young's modulus. The depth-sensing nanoindentation used by Toivola et al. [12] was carried out using loads from 0.1 mN to 3000 mN, whereas for the present study the loads were kept constant at 4 μN. The indentors are obviously very different and the indentation depth is different for the two methods (generally, nanoindentation achieves an indentation depth of 1 μm using materials with a Young's modulus over 1 GPa [25] whereas for the AFM force measurements obtained on uncoated HSQ in the present study, indentation depth was comprised between 6 and 45 nm). Studies on cell attachment have evaluated the adhesion force of different cells to protein coated substrates. They observed that the forces applied by the cells are generally in the range of 100 nN to 1 000 nN. These adhesions forces were estimated at 300 nN to 400 nN for murine fibroblasts [26]. The maximum values were observed for epithelial cells, where the adhesion forces could be as large as 10,000 nN [27]. Therefore, the loads applied using AFM nanoindentation are closer to those applied by cells.

The present study observed an increase in surface roughness with decreasing electron beam doses for developed HSQ, whereas for undeveloped HSQ the roughness values stayed at low levels for the range of electron beam doses studied. The observation of an electron dose dependent roughness of HSQ is confirmed by a previous study by Georgiev et al. [9]. This study found that the surface roughness of HSQ dropped with an increase in the dose. This effect was more pronounced for low electron beam exposures, which is consistent with the present study. The roughness levelled off at about 1 nm around the dose at which the respective contrast curve saturates, so where all the resist thickness is maintained after development. The maximum roughness value observed was about 9 nm, for a pre-bake temperature of 220 °C in TMAH at the onset dose, which is defined as the lowest dose at which HSQ material remains after development. This dose was comprised between 90 μC cm^−2^ and 200 μC cm^−2^, depending on the concentration of TMAH used. For a pre-bake step at 90 °C, Georgiev et al. [9] found that the onset dose increased from 150 μC cm^−2^ to 350 μC cm^−2^, because less cross linking of the HSQ structure had taken place compared to pre-bake at 220 °C. In the present study, the samples were not exposed to any pre-bake. The pre-bake step normally increases the sensitivity of the HSQ to the electron beam, because of the initial cross linking of the structure during this step. This normally leads to a decrease in the onset dose. However, in the present study, the onset dose was around 20 μC cm^−2^, which is much lower than the ones obtained by Georgiev et al. [9] for both 90 °C and 220 °C pre-bake steps. This may be attributed to a different developer used (TMA instead of TMAH). As the pair resist-developer used in the study has a higher sensitivity than the one used by Georgiev et al. [9], less cured HSQ pads with high surface roughness values (up to 40 nm) are maintained after development. The dose dependence of surface roughness observed for HSQ has also been reported in the past for other negative tone electron beam resists [28,29].

Torsional tapping imaging of the Young's modulus of the HSQ samples confirmed the electron beam dose dependent stiffness of HSQ observed using AFM nanoindentation. The distributions of the Young's modulus values were uniform for all HSQ samples studied except for the lowest electron beam exposure of the developed HSQ array, where a second peak was observed at very low Young's modulus values. This might be explained by the fact that the model used for torsional tapping assumes a spherical tip in contact to a flat sample [30]. This approximation is precise only if the tip radius is substantially smaller than the curvature radius of the local topography. In case of high surface roughness, the topography can cause significant changes in contact area which lead to variations in the Young's modulus values calculated. The fact that this variation in stiffness is not observed on the undeveloped sample which is flat confirms that the second peak observed for the developed array at the lowest electron beam exposure could be due to the high surface roughness.

Although dose dependent stiffness variations observed by AFM nanoindentation was confirmed by torsional tapping analysis, the absolute stiffness values differed between the two methods. For the developed sample, torsional tapping mode obtained higher Young's modulus values on the low exposed pads than the force measurements. Also, the sample exposed to 2000 μC cm^−2^ electron beam dose had the highest frequency of stiffness values at about 1 GPa, whereas AFM nanoindentation obtained a value of 0.4 GPa. These differences between the results of AFM nanoindentation and torsional tapping mode could be caused be differences in the force dynamics. Indeed, in AFM nanoindentation, the frequency used was 1 Hz whereas for torsional tapping mode, the frequency is equal to the drive frequency of the cantilever, in this case 55 kHz. In case of a pure elastic response, only a weak frequency dependency is expected and therefore results from the different methods should be comparable. Viscoelastic samples however exhibit distinct changes if the interaction time between the tip and the sample is reduced. A previous study investigated the viscoleastic behaviour of HSQ and observed that temperature curing affected the viscoelasticity of HSQ [31]. At a certain point in the curing, HSQ could be considered as a completely elastic material. This change of viscoelastic behaviour depending on curing would explain the differences observed between torsional tapping mode and AFM nanoindentation. Also, the force loads applied during the torsional tapping mode are around 10 nN, a much smaller load than the one usually used for force measurements, in this case 4000 nN. This leads to a lower indentation into the sample for torsional tapping mode and means that the measurements are more sensitive to possible contamination on the surface or to topography variations. A study focusing on torsional tapping mode imaging found that a large range of material stiffnesses could be studied by torsional tapping mode but for compliant materials with modulus values in the MegaPascals range, the standard deviation of modulus measurements increased up to 50% of the nominal value [32].

The Young's modulus of HSQ increased after oxygen plasma exposure. Previous studies have observed that the physical properties of HSQ are modified following oxygen plasma exposure [33–35]. Penaud *et al.* observed that oxygen plasma treatment caused transformation of the uncured cage structure to the network structure, which is an indication of an increased crosslinked structure [36]. The change in the cross linking ratio of HSQ was dependent on the power used for treatment and on the treatment time. The increase in the cross linking ratio is therefore responsible for the increase in Young's modulus observed in the present study following oxygen plasma treatment.

After deposition of ppAAm, the modulus was decreased for both HSQ arrays. However, stiffness variation was still observed across the array depending on the electron beam dose used for curing. This suggests that the stiffness measured is a combination of the stiffness of the ppAAm film and the stiffness of the underlying HSQ. A previous study investigating the effect on Young's modulus measurements of a substrate under a thin film during nanoindentation found that, when the Young's modulus of the thin film was smaller than that of its substrate, the calculated Young's modulus of the thin film increased with the indenter maximum displacement. The effect of the hard substrate on the Young's modulus measurements was visible when the indentation was over 10% of the film thickness [37]. Another study using nanoindentation on plasma polymerised hexane films of 1500 nm thickness found that, for indentation depths less than 400 nm, the measured Young's modulus represents the properties from the deposited film only. The influence of the underlying substrate on the composite indentation response is felt for higher indentation depths [38]. In the present study, indentation into the ppAAm coated HSQ ranged from 33 to 52 nm with a ppAAm thickness estimated at 30 nm, which suggests an influence of the stiffness of HSQ on the measured Young's modulus values. In addition, the effect of different thicknesses of ppAAM films deposited on silicon was also investigated and it was shown that the Young's modulus values measured by AFM nanoindentation decreased with increasing ppAAm film thickness. At the ppAAm thickness used in this study (30 nm), the measured stiffness values were much higher than for thicker ppAAm films although the Young's modulus values decreased compared to uncoated silicon. This shows that for 30 nm ppAAm films deposited on a harder substrate, the underlying substrate stiffness is still taken into account by AFM nanoindentation.

Mesenchymal stem cells were cultured on the ppAAm coated HSQ arrays for 7 days, although under these conditions, the cells did not show a significant trend in morphology with HSQ pad stiffness. This may be because the range of stiffness achieved in this study (between 0.1 GPa and 1 GPa) is higher than the one used by Engler et al. (between 1 kPa to 100 kPa) to show the effect of stiffness variations on mesenchymal stem cell behaviour [1]. The effect of stiffer surfaces on stem cell differentiation requires further investigation.

## 5 Conclusions

In the present study, it was observed that electron beam curing of HSQ is able to control the surface Young's modulus over a large range of stiffness values, with good resolution. Electron beam curing also allowed the creation of two dimensional matrices, with highly controlled feature geometry and spatial distribution.

The exposure dose dependency of HSQ Young's modulus and surface roughness was studied with or without development in TMA, with the data indicating an increase in Young's modulus with electron beam exposure for both developed and undeveloped HSQ samples. For developed HSQ, the greater the electron dose, the smoother the sample, whereas, without development, the RMS roughness values were low across the exposure range studied. The HSQ stiffness could therefore be effectively controlled via the electron beam dose used for curing.

Mesenchymal stem cells were cultured on the HSQ samples after coating with ppAAm illustrating the potential of this system to spatially control cell response.
